# Crystal structure of 12-benzyl­sulfanyl-2,9-di­bromo-6*H*-dibenzo[*b*,*g*][1,8]naphthyridin-11-one

**DOI:** 10.1107/S2056989015014541

**Published:** 2015-08-22

**Authors:** Sebastian Resch, Thomas Quell, Dieter Schollmeyer, Siegfried R. Waldvogel

**Affiliations:** a Johannes Gutenberg-University Mainz, Duesbergweg 10-14, 55099 Mainz, Germany

**Keywords:** crystal structure, 1,8-naphthyridine, hetero­tetra­cene

## Abstract

The hetero­tetra­cene skeleton of the title mol­ecule, C_23_H_14_Br_2_N_2_OS, is defined by linear annulation of four six-membered rings, including two N heteroatoms. This moiety is nearly planar (r.m.s. deviation = 0.055 Å), with a slight twist of 4.1 (2)° between the two halves of the aromatic system. The dihedral angle between the least-squares plane of the skeleton and the benzyl group is 24.5 (3)°; the C—S—C angle involving the benzyl­sulfanyl group is 99.2 (4)°. In the crystal, mol­ecules are π-stacked in an anti­parallel fashion along [110], with a distance between the aromatic planes of 3.47 (2) Å. Inter­molecular N—H⋯O hydrogen bonds form chains extending parallel to [001] and bridge the anti­parallel inter­digitated stacks of mol­ecules.

## Related literature   

The title compound was prepared as part of a study towards sulfur-containing 1,8-naphthyridine derivatives (Resch *et al.*, 2015 ▸) in which the structure of a dibenzo[*b*,*g*][1,2]di­thiolo[3,4,5-*d*,*e*][1,8]naphthyridine derivative is reported. For the structure of tetra­cene, see: Holmes *et al.* (1999 ▸).
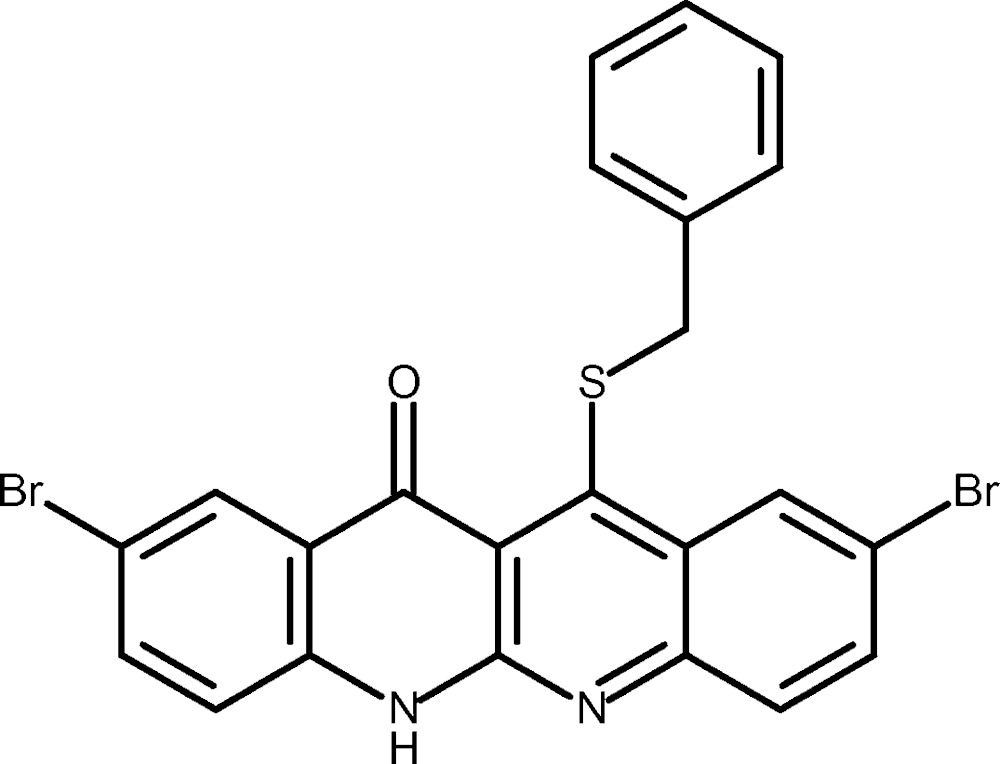


## Experimental   

### Crystal data  

C_23_H_14_Br_2_N_2_OS
*M* *_r_* = 526.24
Monoclinic, 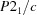

*a* = 15.4915 (10) Å
*b* = 9.3953 (4) Å
*c* = 13.6501 (9) Å
β = 101.251 (5)°
*V* = 1948.6 (2) Å^3^
*Z* = 4
Mo *K*α radiation
μ = 4.29 mm^−1^
*T* = 193 K
0.27 × 0.12 × 0.04 mm

### Data collection  

Stoe IPDS 2T diffractometer
Absorption correction: integration *X-RED* (Stoe & Cie, 1995 ▸) *T*_min_ = 0.363, *T*_max_ = 0.811
10437 measured reflections
4807 independent reflections
2850 reflections with *I* > 2σ(*I*)
*R*_int_ = 0.058

### Refinement  

*R*[*F* ^2^ > 2σ(*F* ^2^)] = 0.068
*wR*(*F* ^2^) = 0.222
*S* = 1.05
4807 reflections
262 parameters
H-atom parameters constrained
Δρ_max_ = 2.29 e Å^−3^
Δρ_min_ = −1.21 e Å^−3^

### 

Data collection: *X-AREA* (Stoe & Cie, 1995 ▸); cell refinement: *X-AREA*; data reduction: *X-RED* (Stoe & Cie, 1995 ▸); program(s) used to solve structure: *SIR2004* (Burla *et al.*, 2005 ▸); program(s) used to refine structure: *SHELXL2014* (Sheldrick, 2015 ▸); molecular graphics: *PLATON* (Spek, 2009 ▸); software used to prepare material for publication: *publCIF* (Westrip, 2010 ▸).

## Supplementary Material

Crystal structure: contains datablock(s) I, New_Global_Publ_Block. DOI: 10.1107/S2056989015014541/wm5191sup1.cif

Structure factors: contains datablock(s) I. DOI: 10.1107/S2056989015014541/wm5191Isup2.hkl

Supporting information file. DOI: 10.1107/S2056989015014541/wm5191Isup3.cml

**Fig. 1.**
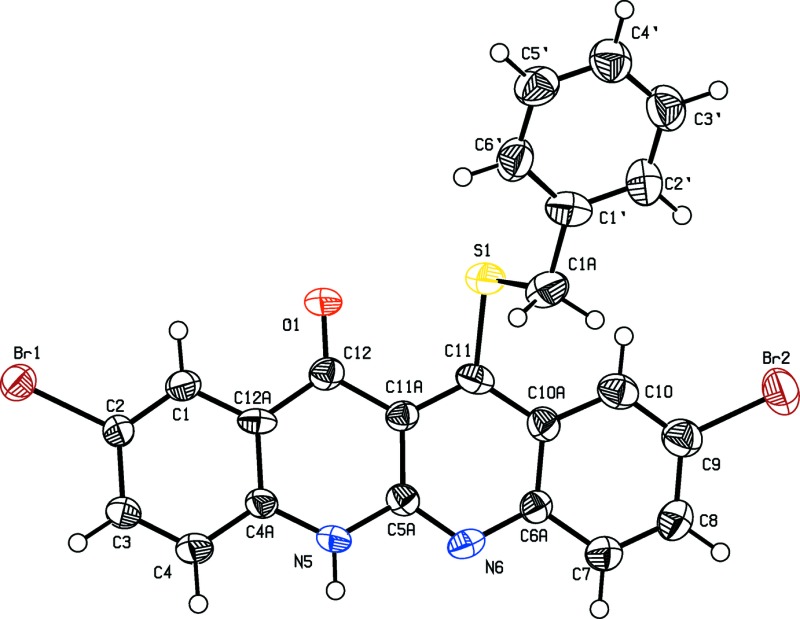
The molecular structure of the title compound with labeling and displacement ellipsoids drawn at the 50% probability level.

. DOI: 10.1107/S2056989015014541/wm5191fig1.tif
The mol­ecular structure of the title compound with labeling and displacement ellipsoids drawn at the 50% probability level.

**Fig. 2.**
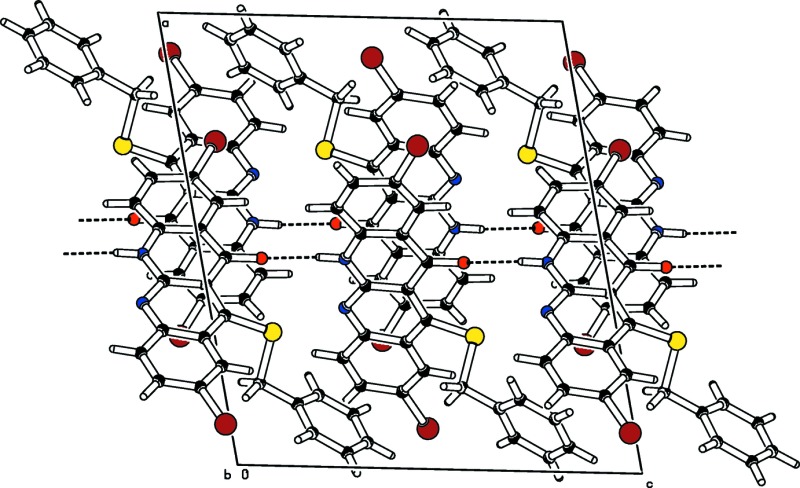
The crystal structure of the title compound in a view along [010]. N—H···O hydrogen bonds are shown as dashed lines.

. DOI: 10.1107/S2056989015014541/wm5191fig2.tif
The crystal structure of the title compound in a view along [010]. N—H⋯O hydrogen bonds are shown as dashed lines.

CCDC reference: 1416554

Additional supporting information:  crystallographic information; 3D view; checkCIF report

## Figures and Tables

**Table 1:** Hydrogen-bond geometry (, )

*D*H*A*	*D*H	H*A*	*D* *A*	*D*H*A*
N5H5O1^i^	0.88	2.28	3.001(7)	140

Symmetry code: (i) 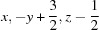.

## supplementary crystallographic information

### S1. Synthesis and crystallization

In argon atmosphere, 50 mg (2.0 mmol, 3 eq) NaH and 340 mg (2.7 mmol, 4 eq) benzyl thiol were given to 40 ml of anhydrous dioxane and stirred at room temperature for 30 min. 300 mg (0.68 mmol, 1 eq) of 6*H*-12-chloro-2,9-di­bromo­dibenzo[*b,g*]-1,8-naphthyridin-11-one were added and the mixture stirred at room temperature for additional 5 h. After completion of the reaction, the solvent was removed under reduced pressure and the residue purified by column chromatography on silica gel using a mixture of di­chloro­methane and acetic acid (97:3). The solvent was removed under reduced pressure and the residue alkalized using 1*M* ammonium hydroxide solution. Yield: 276 mg (0.52 mmol, 77%) of an orange solid with mp. = > 513 K (decomposition). Suitable single crystals were obtained by slowly diluting a saturated solution of the title compound in di­chloro­methane/methanol (5:1) by cyclo­hexane (diffusion method).

### S2. Refinement

Hydrogen atoms attached to carbon atoms were placed at calculated positions with C—H = 0.95 Å (aromatic) or 0.99 Å (methyl­ene C atom). The H atom bonded to the N atom was placed at calculated positions with N—H = 0.88 Å. All H atoms were refined in the riding-model approximation with isotropic displacement parameters (set at 1.2–1.5*U*_eq_ of the parent atom).

### Crystal data

**Table:**

C_23_H_14_Br_2_N_2_OS	*F*(000) = 1040
*M**_r_* = 526.24	*D*_x_ = 1.794 Mg m^−^^3^
Monoclinic, *P*2_1_/*c*	Mo *K*α radiation, λ = 0.71073 Å
*a* = 15.4915 (10) Å	Cell parameters from 7178 reflections
*b* = 9.3953 (4) Å	θ = 2.6–28.2°
*c* = 13.6501 (9) Å	µ = 4.29 mm^−^^1^
β = 101.251 (5)°	*T* = 193 K
*V* = 1948.6 (2) Å^3^	Plate, orange
*Z* = 4	0.27 × 0.12 × 0.04 mm

### Data collection

**Table:**

Stoe IPDS 2T diffractometer	4807 independent reflections
Radiation source: sealed X-ray tube, 12 x 0.4 mm long-fine focus	2850 reflections with *I* > 2σ(*I*)
Detector resolution: 6.67 pixels mm^-1^	*R*_int_ = 0.058
rotation method scans	θ_max_ = 28.3°, θ_min_ = 2.6°
Absorption correction: integration *X-RED* (Stoe & Cie, 1995)	*h* = −20→20
*T*_min_ = 0.363, *T*_max_ = 0.811	*k* = −10→12
10437 measured reflections	*l* = −18→16

### Refinement

**Table:**

Refinement on *F*^2^	0 restraints
Least-squares matrix: full	Hydrogen site location: inferred from neighbouring sites
*R*[*F*^2^ > 2σ(*F*^2^)] = 0.068	H-atom parameters constrained
*wR*(*F*^2^) = 0.222	*w* = 1/[σ^2^(*F*_o_^2^) + (0.1106*P*)^2^ + 4.6375*P*] where *P* = (*F*_o_^2^ + 2*F*_c_^2^)/3
*S* = 1.05	(Δ/σ)_max_ = 0.001
4807 reflections	Δρ_max_ = 2.29 e Å^−^^3^
262 parameters	Δρ_min_ = −1.21 e Å^−^^3^

### Special details

**Table:**

Geometry. All e.s.d.'s (except the e.s.d. in the dihedral angle between two l.s. planes) are estimated using the full covariance matrix. The cell e.s.d.'s are taken into account individually in the estimation of e.s.d.'s in distances, angles and torsion angles; correlations between e.s.d.'s in cell parameters are only used when they are defined by crystal symmetry. An approximate (isotropic) treatment of cell e.s.d.'s is used for estimating e.s.d.'s involving l.s. planes.

### Fractional atomic coordinates and isotropic or equivalent isotropic displacement parameters (Å^2^)

**Table:**

	*x*	*y*	*z*	*U*_iso_*/*U*_eq_	
Br1	0.71684 (5)	1.08811 (8)	0.58620 (5)	0.0411 (2)	
Br2	0.08972 (7)	0.12934 (12)	0.48824 (9)	0.0689 (3)	
S1	0.29644 (15)	0.5906 (3)	0.64477 (16)	0.0589 (6)	
O1	0.4597 (3)	0.7273 (6)	0.6495 (3)	0.0438 (12)	
C1	0.5809 (5)	0.8812 (7)	0.5634 (5)	0.0348 (14)	
H1	0.5838	0.8954	0.6329	0.042*	
C2	0.6354 (4)	0.9552 (7)	0.5144 (5)	0.0328 (13)	
C3	0.6323 (5)	0.9381 (8)	0.4114 (5)	0.0377 (15)	
H3	0.6694	0.9933	0.3785	0.045*	
C4	0.5755 (5)	0.8415 (8)	0.3590 (5)	0.0390 (16)	
H4	0.5736	0.8280	0.2897	0.047*	
C4A	0.5201 (4)	0.7624 (8)	0.4081 (5)	0.0322 (14)	
N5	0.4632 (4)	0.6622 (6)	0.3565 (4)	0.0355 (12)	
H5	0.4652	0.6476	0.2933	0.043*	
C5A	0.4042 (4)	0.5846 (7)	0.3968 (5)	0.0301 (13)	
N6	0.3543 (4)	0.4954 (6)	0.3354 (4)	0.0347 (12)	
C6A	0.2948 (4)	0.4160 (7)	0.3729 (5)	0.0315 (13)	
C7	0.2433 (5)	0.3168 (8)	0.3085 (5)	0.0409 (16)	
H7	0.2516	0.3067	0.2417	0.049*	
C8	0.1822 (5)	0.2364 (8)	0.3411 (6)	0.0451 (17)	
H8	0.1472	0.1713	0.2970	0.054*	
C9	0.1706 (5)	0.2493 (9)	0.4408 (6)	0.0477 (18)	
C10	0.2184 (5)	0.3449 (8)	0.5041 (6)	0.0425 (16)	
H10	0.2094	0.3526	0.5708	0.051*	
C10A	0.2809 (4)	0.4321 (7)	0.4720 (5)	0.0353 (14)	
C11	0.3307 (4)	0.5375 (8)	0.5332 (5)	0.0361 (14)	
C11A	0.3987 (4)	0.6075 (7)	0.4993 (5)	0.0293 (13)	
C12	0.4597 (4)	0.7092 (7)	0.5603 (5)	0.0335 (14)	
C12A	0.5203 (4)	0.7839 (7)	0.5102 (4)	0.0319 (14)	
C1A	0.1824 (6)	0.6501 (11)	0.5902 (6)	0.057 (2)	
H1AA	0.1841	0.7334	0.5462	0.068*	
H1AB	0.1497	0.5726	0.5500	0.068*	
C1'	0.1381 (6)	0.6890 (10)	0.6749 (6)	0.0500 (19)	
C2'	0.0739 (6)	0.6017 (10)	0.6987 (7)	0.055 (2)	
H2'	0.0582	0.5173	0.6612	0.066*	
C3'	0.0318 (6)	0.6350 (11)	0.7769 (7)	0.058 (2)	
H3'	−0.0128	0.5746	0.7925	0.070*	
C4'	0.0557 (6)	0.7568 (10)	0.8311 (7)	0.055 (2)	
H4'	0.0282	0.7796	0.8855	0.066*	
C5'	0.1175 (6)	0.8441 (10)	0.8082 (7)	0.054 (2)	
H5'	0.1328	0.9281	0.8464	0.065*	
C6'	0.1586 (6)	0.8136 (9)	0.7307 (7)	0.052 (2)	
H6'	0.2014	0.8774	0.7146	0.062*	

### Atomic displacement parameters (Å^2^)

**Table:**

	*U*^11^	*U*^22^	*U*^33^	*U*^12^	*U*^13^	*U*^23^
Br1	0.0434 (4)	0.0406 (4)	0.0375 (4)	−0.0045 (3)	0.0034 (3)	−0.0001 (3)
Br2	0.0683 (6)	0.0662 (6)	0.0812 (7)	−0.0265 (5)	0.0367 (5)	−0.0144 (5)
S1	0.0482 (11)	0.0958 (18)	0.0361 (10)	−0.0175 (11)	0.0165 (8)	−0.0164 (10)
O1	0.048 (3)	0.062 (3)	0.022 (2)	−0.011 (3)	0.008 (2)	−0.002 (2)
C1	0.034 (3)	0.041 (4)	0.028 (3)	0.001 (3)	0.005 (3)	−0.004 (3)
C2	0.032 (3)	0.037 (3)	0.029 (3)	−0.005 (3)	0.005 (3)	0.001 (3)
C3	0.043 (4)	0.043 (4)	0.027 (3)	−0.006 (3)	0.006 (3)	0.006 (3)
C4	0.042 (4)	0.049 (4)	0.025 (3)	−0.002 (3)	0.004 (3)	0.003 (3)
C4A	0.028 (3)	0.044 (4)	0.024 (3)	−0.002 (3)	0.004 (2)	0.003 (3)
N5	0.039 (3)	0.047 (3)	0.022 (3)	−0.005 (3)	0.010 (2)	0.001 (2)
C5A	0.031 (3)	0.030 (3)	0.028 (3)	0.003 (3)	0.004 (2)	0.007 (2)
N6	0.038 (3)	0.038 (3)	0.027 (3)	0.004 (2)	0.003 (2)	−0.008 (2)
C6A	0.025 (3)	0.035 (3)	0.033 (3)	0.003 (3)	0.004 (2)	−0.002 (3)
C7	0.044 (4)	0.043 (4)	0.035 (4)	−0.002 (3)	0.007 (3)	0.000 (3)
C8	0.044 (4)	0.039 (4)	0.050 (4)	−0.004 (3)	0.005 (3)	−0.007 (3)
C9	0.048 (4)	0.050 (5)	0.047 (4)	0.001 (3)	0.015 (4)	−0.002 (3)
C10	0.040 (4)	0.049 (4)	0.040 (4)	0.008 (3)	0.012 (3)	0.002 (3)
C10A	0.031 (3)	0.038 (4)	0.038 (4)	−0.001 (3)	0.009 (3)	−0.001 (3)
C11	0.035 (3)	0.046 (4)	0.029 (3)	0.006 (3)	0.011 (3)	0.003 (3)
C11A	0.028 (3)	0.033 (3)	0.026 (3)	0.003 (2)	0.003 (2)	0.000 (2)
C12	0.032 (3)	0.038 (4)	0.028 (3)	0.003 (3)	0.000 (3)	0.003 (3)
C12A	0.038 (3)	0.039 (4)	0.019 (3)	0.009 (3)	0.007 (2)	0.003 (2)
C1A	0.055 (5)	0.075 (6)	0.039 (4)	−0.007 (4)	0.004 (4)	−0.011 (4)
C1'	0.056 (5)	0.059 (5)	0.037 (4)	0.010 (4)	0.013 (3)	0.004 (3)
C2'	0.047 (5)	0.053 (5)	0.067 (6)	−0.010 (4)	0.013 (4)	0.000 (4)
C3'	0.042 (4)	0.069 (6)	0.063 (6)	−0.005 (4)	0.012 (4)	0.004 (5)
C4'	0.044 (4)	0.064 (6)	0.058 (5)	0.003 (4)	0.011 (4)	0.001 (4)
C5'	0.053 (5)	0.057 (5)	0.054 (5)	0.006 (4)	0.013 (4)	−0.016 (4)
C6'	0.052 (5)	0.045 (4)	0.058 (5)	−0.010 (4)	0.008 (4)	−0.008 (4)

### Geometric parameters (Å, º)

**Table:**

Br1—C2	1.905 (7)	C8—C9	1.413 (11)
Br2—C9	1.890 (9)	C8—H8	0.9500
S1—C11	1.779 (7)	C9—C10	1.362 (11)
S1—C1A	1.864 (9)	C10—C10A	1.402 (10)
O1—C12	1.229 (8)	C10—H10	0.9500
C1—C2	1.366 (10)	C10A—C11	1.422 (10)
C1—C12A	1.406 (10)	C11—C11A	1.395 (10)
C1—H1	0.9500	C11A—C12	1.481 (9)
C2—C3	1.407 (9)	C12—C12A	1.447 (10)
C3—C4	1.365 (10)	C1A—C1'	1.501 (12)
C3—H3	0.9500	C1A—H1AA	0.9900
C4—C4A	1.400 (10)	C1A—H1AB	0.9900
C4—H4	0.9500	C1'—C2'	1.376 (12)
C4A—N5	1.385 (9)	C1'—C6'	1.398 (12)
C4A—C12A	1.408 (9)	C2'—C3'	1.391 (13)
N5—C5A	1.366 (8)	C2'—H2'	0.9500
N5—H5	0.8800	C3'—C4'	1.374 (13)
C5A—N6	1.323 (8)	C3'—H3'	0.9500
C5A—C11A	1.435 (9)	C4'—C5'	1.342 (13)
N6—C6A	1.360 (9)	C4'—H4'	0.9500
C6A—C7	1.416 (10)	C5'—C6'	1.368 (12)
C6A—C10A	1.420 (10)	C5'—H5'	0.9500
C7—C8	1.352 (11)	C6'—H6'	0.9500
C7—H7	0.9500		
			
C11—S1—C1A	99.2 (4)	C10—C10A—C11	123.5 (7)
C2—C1—C12A	119.5 (6)	C6A—C10A—C11	117.9 (6)
C2—C1—H1	120.3	C11A—C11—C10A	119.5 (6)
C12A—C1—H1	120.3	C11A—C11—S1	121.5 (5)
C1—C2—C3	121.8 (6)	C10A—C11—S1	118.6 (5)
C1—C2—Br1	119.5 (5)	C11—C11A—C5A	116.4 (6)
C3—C2—Br1	118.7 (5)	C11—C11A—C12	123.9 (6)
C4—C3—C2	119.5 (6)	C5A—C11A—C12	119.5 (6)
C4—C3—H3	120.2	O1—C12—C12A	121.8 (6)
C2—C3—H3	120.2	O1—C12—C11A	121.5 (6)
C3—C4—C4A	119.7 (6)	C12A—C12—C11A	116.7 (6)
C3—C4—H4	120.2	C1—C12A—C4A	118.5 (6)
C4A—C4—H4	120.2	C1—C12A—C12	120.0 (6)
N5—C4A—C4	120.4 (6)	C4A—C12A—C12	121.5 (6)
N5—C4A—C12A	118.6 (6)	C1'—C1A—S1	107.8 (6)
C4—C4A—C12A	120.9 (6)	C1'—C1A—H1AA	110.1
C5A—N5—C4A	124.6 (6)	S1—C1A—H1AA	110.1
C5A—N5—H5	117.7	C1'—C1A—H1AB	110.1
C4A—N5—H5	117.7	S1—C1A—H1AB	110.1
N6—C5A—N5	116.0 (6)	H1AA—C1A—H1AB	108.5
N6—C5A—C11A	125.1 (6)	C2'—C1'—C6'	117.9 (8)
N5—C5A—C11A	119.0 (6)	C2'—C1'—C1A	119.8 (8)
C5A—N6—C6A	117.7 (6)	C6'—C1'—C1A	122.3 (8)
N6—C6A—C7	117.9 (6)	C1'—C2'—C3'	121.1 (9)
N6—C6A—C10A	122.7 (6)	C1'—C2'—H2'	119.5
C7—C6A—C10A	119.3 (6)	C3'—C2'—H2'	119.5
C8—C7—C6A	120.6 (7)	C4'—C3'—C2'	118.8 (9)
C8—C7—H7	119.7	C4'—C3'—H3'	120.6
C6A—C7—H7	119.7	C2'—C3'—H3'	120.6
C7—C8—C9	119.9 (7)	C5'—C4'—C3'	120.9 (9)
C7—C8—H8	120.0	C5'—C4'—H4'	119.5
C9—C8—H8	120.0	C3'—C4'—H4'	119.5
C10—C9—C8	120.8 (8)	C4'—C5'—C6'	120.8 (9)
C10—C9—Br2	119.4 (6)	C4'—C5'—H5'	119.6
C8—C9—Br2	119.8 (6)	C6'—C5'—H5'	119.6
C9—C10—C10A	120.7 (7)	C5'—C6'—C1'	120.4 (8)
C9—C10—H10	119.6	C5'—C6'—H6'	119.8
C10A—C10—H10	119.6	C1'—C6'—H6'	119.8
C10—C10A—C6A	118.5 (6)		
			
C12A—C1—C2—C3	0.7 (11)	C10A—C11—C11A—C5A	10.1 (9)
C12A—C1—C2—Br1	179.5 (5)	S1—C11—C11A—C5A	−162.6 (5)
C1—C2—C3—C4	−2.3 (11)	C10A—C11—C11A—C12	−174.3 (6)
Br1—C2—C3—C4	178.8 (6)	S1—C11—C11A—C12	13.0 (9)
C2—C3—C4—C4A	1.0 (11)	N6—C5A—C11A—C11	−5.1 (10)
C3—C4—C4A—N5	−178.9 (7)	N5—C5A—C11A—C11	172.9 (6)
C3—C4—C4A—C12A	1.9 (11)	N6—C5A—C11A—C12	179.0 (6)
C4—C4A—N5—C5A	−177.0 (6)	N5—C5A—C11A—C12	−2.9 (9)
C12A—C4A—N5—C5A	2.2 (10)	C11—C11A—C12—O1	7.7 (10)
C4A—N5—C5A—N6	179.0 (6)	C5A—C11A—C12—O1	−176.8 (6)
C4A—N5—C5A—C11A	0.8 (10)	C11—C11A—C12—C12A	−173.5 (6)
N5—C5A—N6—C6A	179.9 (6)	C5A—C11A—C12—C12A	2.0 (9)
C11A—C5A—N6—C6A	−2.0 (9)	C2—C1—C12A—C4A	2.2 (10)
C5A—N6—C6A—C7	−177.9 (6)	C2—C1—C12A—C12	−177.5 (6)
C5A—N6—C6A—C10A	4.1 (9)	N5—C4A—C12A—C1	177.3 (6)
N6—C6A—C7—C8	−179.0 (7)	C4—C4A—C12A—C1	−3.5 (10)
C10A—C6A—C7—C8	−1.0 (11)	N5—C4A—C12A—C12	−3.1 (10)
C6A—C7—C8—C9	−1.1 (12)	C4—C4A—C12A—C12	176.2 (6)
C7—C8—C9—C10	1.9 (12)	O1—C12—C12A—C1	−0.5 (10)
C7—C8—C9—Br2	−176.6 (6)	C11A—C12—C12A—C1	−179.4 (6)
C8—C9—C10—C10A	−0.5 (12)	O1—C12—C12A—C4A	179.8 (7)
Br2—C9—C10—C10A	178.0 (6)	C11A—C12—C12A—C4A	1.0 (9)
C9—C10—C10A—C6A	−1.7 (11)	C11—S1—C1A—C1'	175.4 (7)
C9—C10—C10A—C11	177.7 (7)	S1—C1A—C1'—C2'	−107.4 (8)
N6—C6A—C10A—C10	−179.7 (6)	S1—C1A—C1'—C6'	73.6 (10)
C7—C6A—C10A—C10	2.4 (10)	C6'—C1'—C2'—C3'	−1.0 (14)
N6—C6A—C10A—C11	0.9 (10)	C1A—C1'—C2'—C3'	179.9 (8)
C7—C6A—C10A—C11	−177.0 (6)	C1'—C2'—C3'—C4'	−0.5 (14)
C10—C10A—C11—C11A	172.4 (7)	C2'—C3'—C4'—C5'	1.3 (14)
C6A—C10A—C11—C11A	−8.3 (10)	C3'—C4'—C5'—C6'	−0.4 (14)
C10—C10A—C11—S1	−14.7 (10)	C4'—C5'—C6'—C1'	−1.2 (14)
C6A—C10A—C11—S1	164.7 (5)	C2'—C1'—C6'—C5'	1.9 (13)
C1A—S1—C11—C11A	115.4 (6)	C1A—C1'—C6'—C5'	−179.1 (8)
C1A—S1—C11—C10A	−57.4 (7)		

### Hydrogen-bond geometry (Å, º)

**Table:**

*D*—H···*A*	*D*—H	H···*A*	*D*···*A*	*D*—H···*A*
N5—H5···O1^i^	0.88	2.28	3.001 (7)	140

Symmetry code: (i) *x*, −*y*+3/2, *z*−1/2.
