# Supplementary figures and images for: Crystal structure of 12-benzyl­sulfanyl-2,9-di­bromo-6H-dibenzo[b,g][1,8]naphthyridin-11-one
Source: Acta Crystallogr E Crystallogr Commun. 2015 Aug 22;71(Pt 9):o675–6. doi: 10.1107/S2056989015014541 (PMC4555377; doi:10.1107/S2056989015014541)

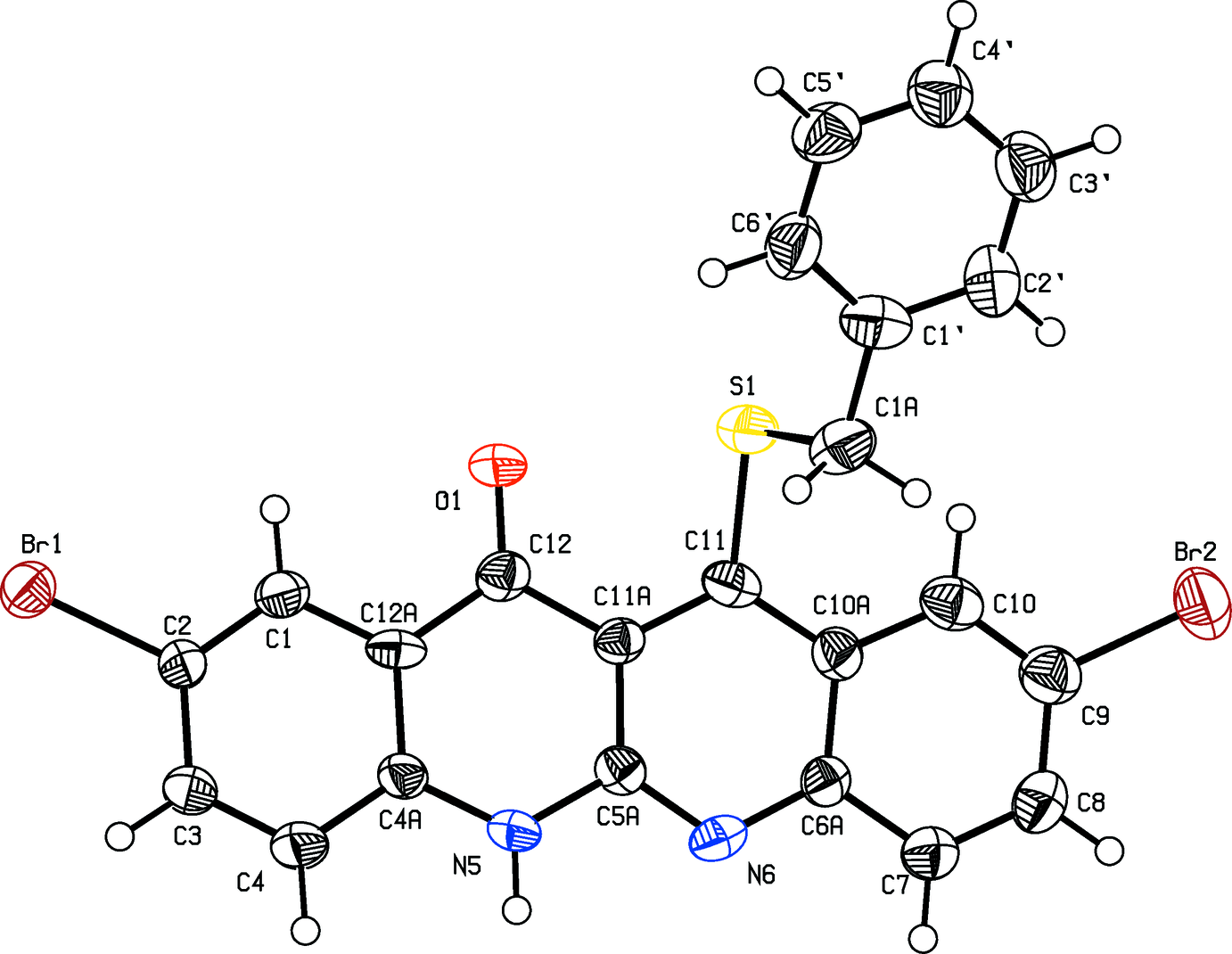

Supplement: Supplementary file 4 [file e-71-0o675-fig1.tif]

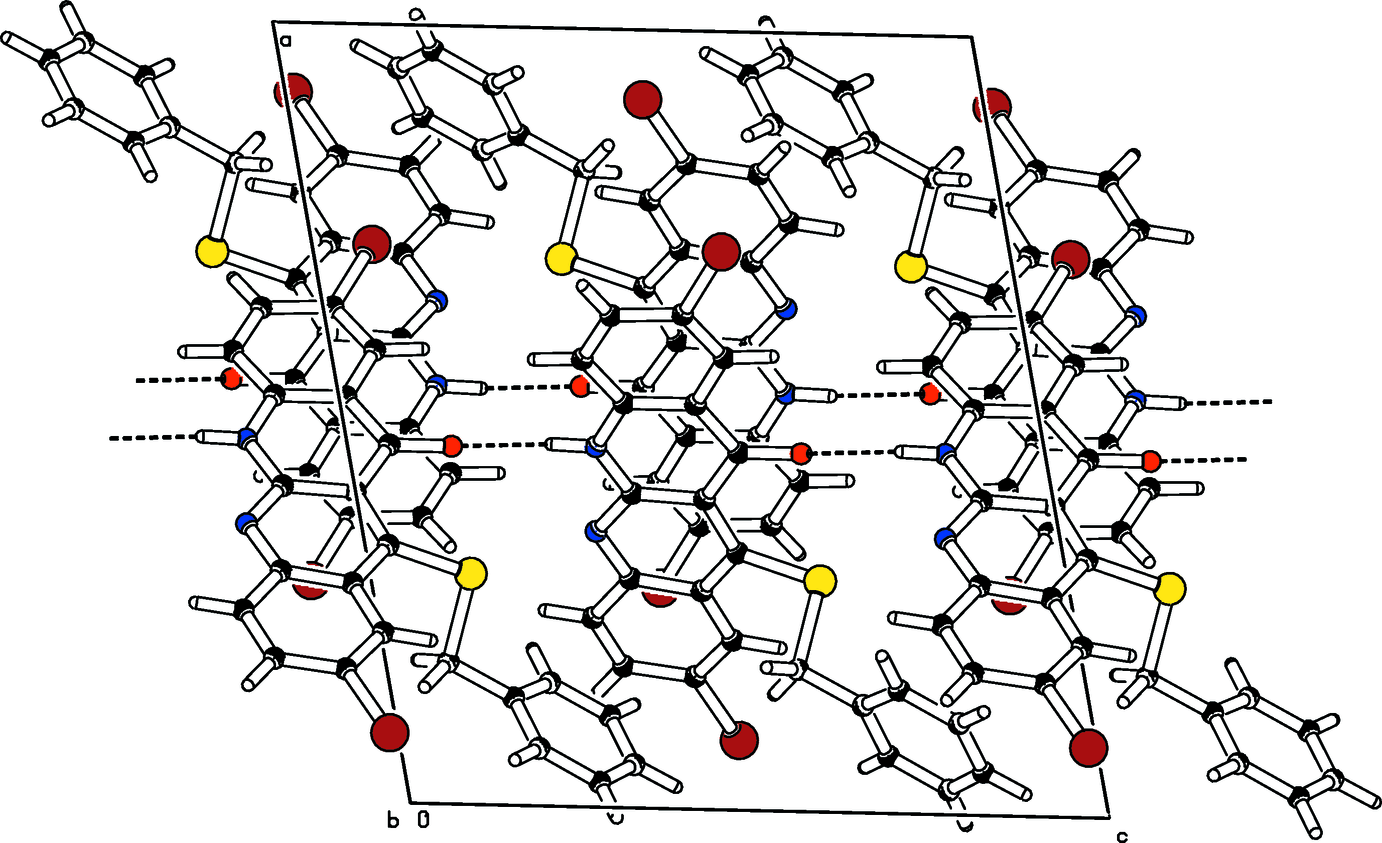

Supplement: Supplementary file 5 [file e-71-0o675-fig2.tif]
